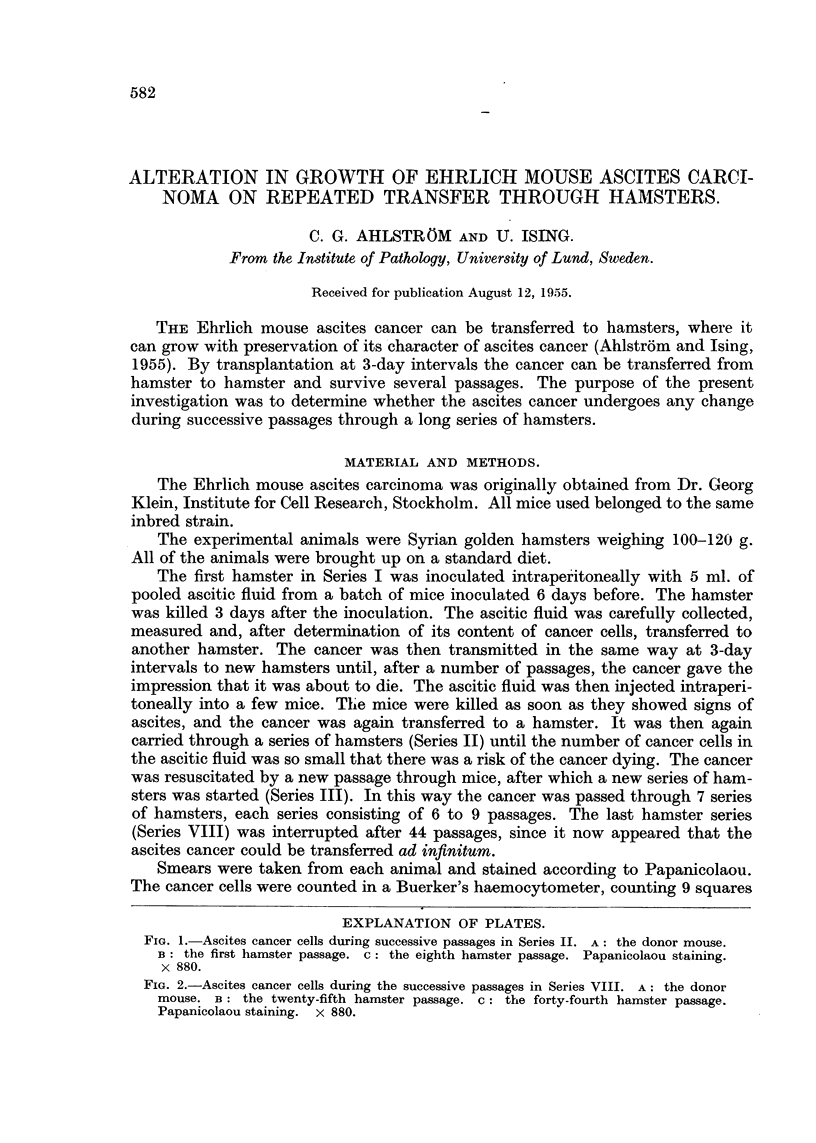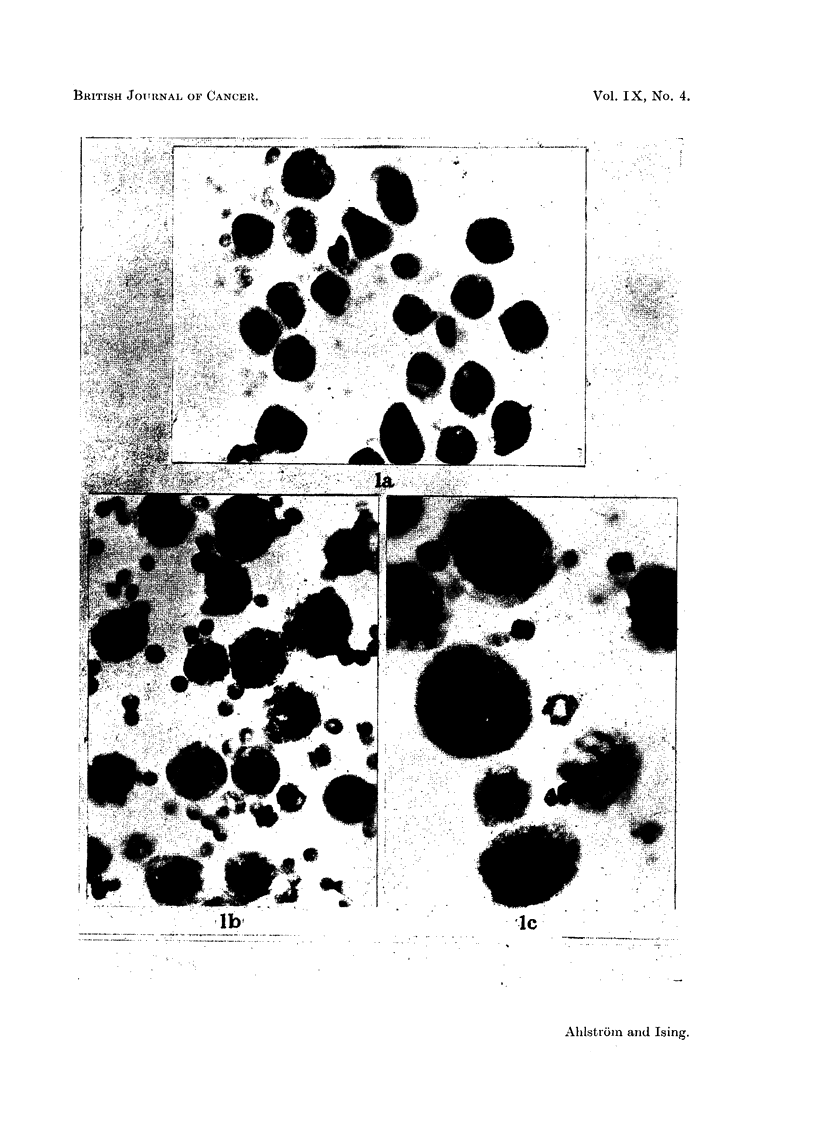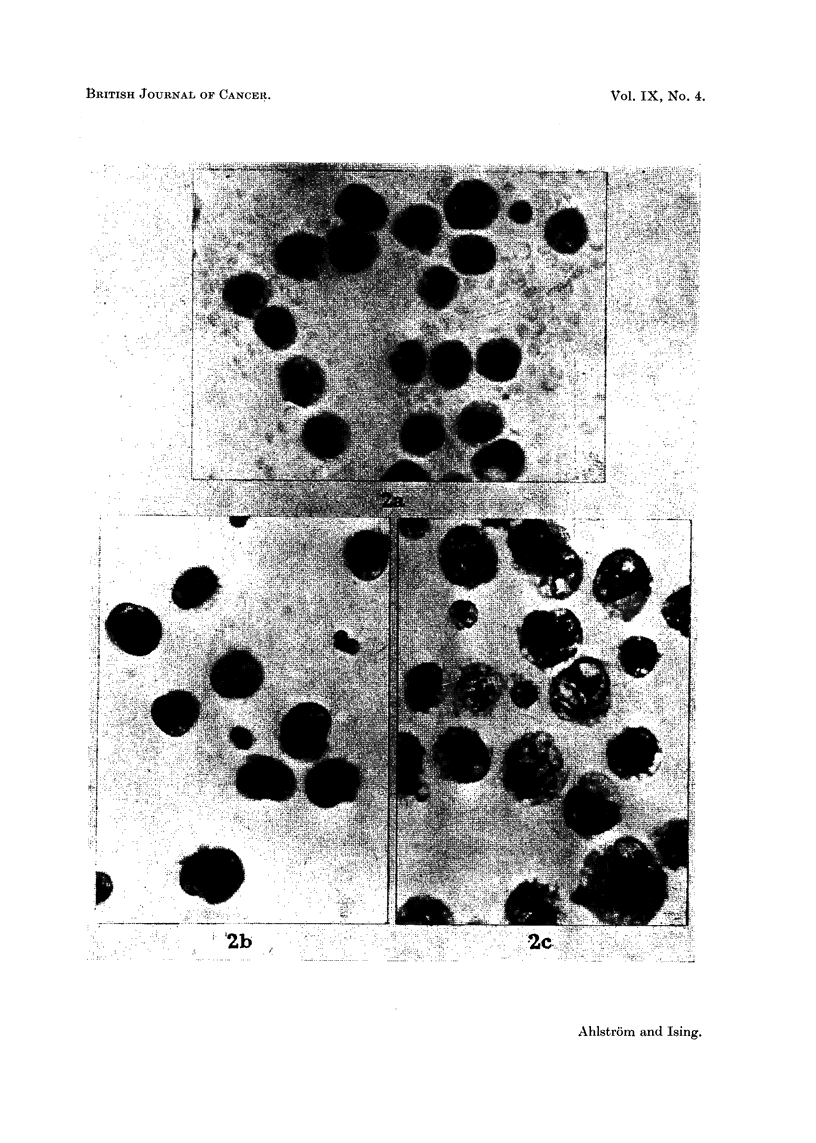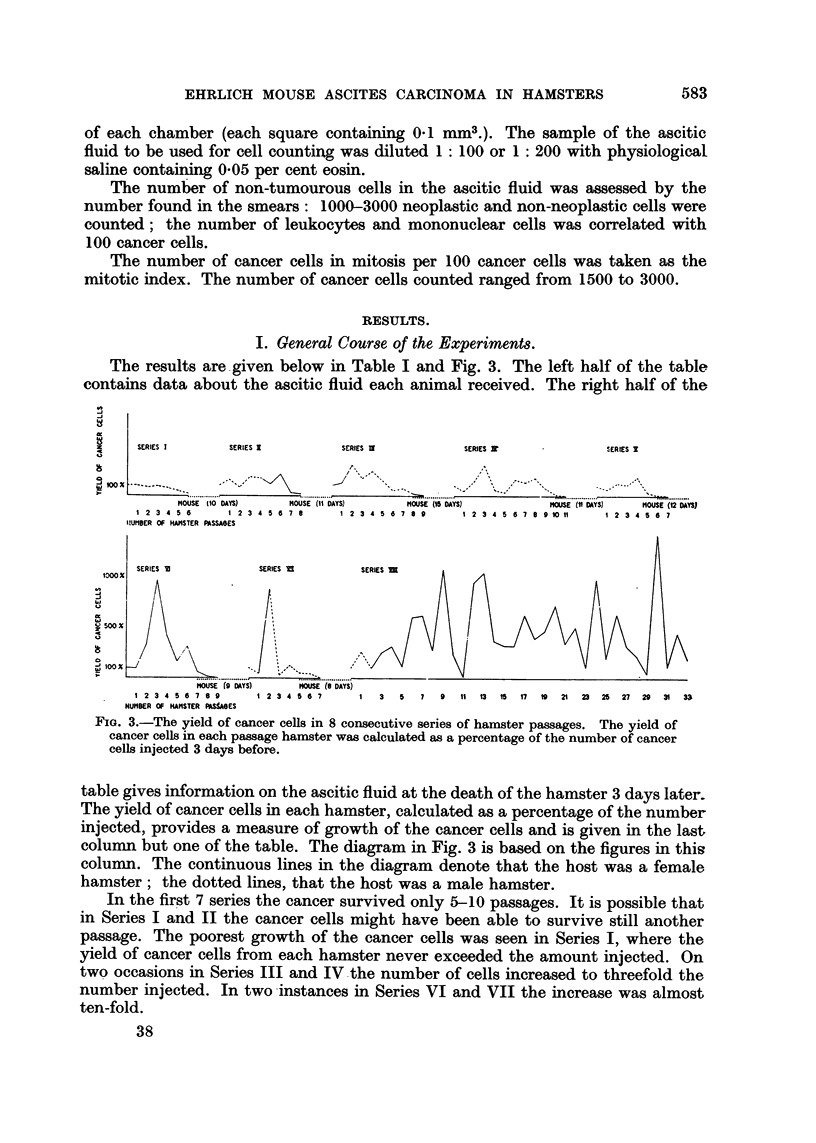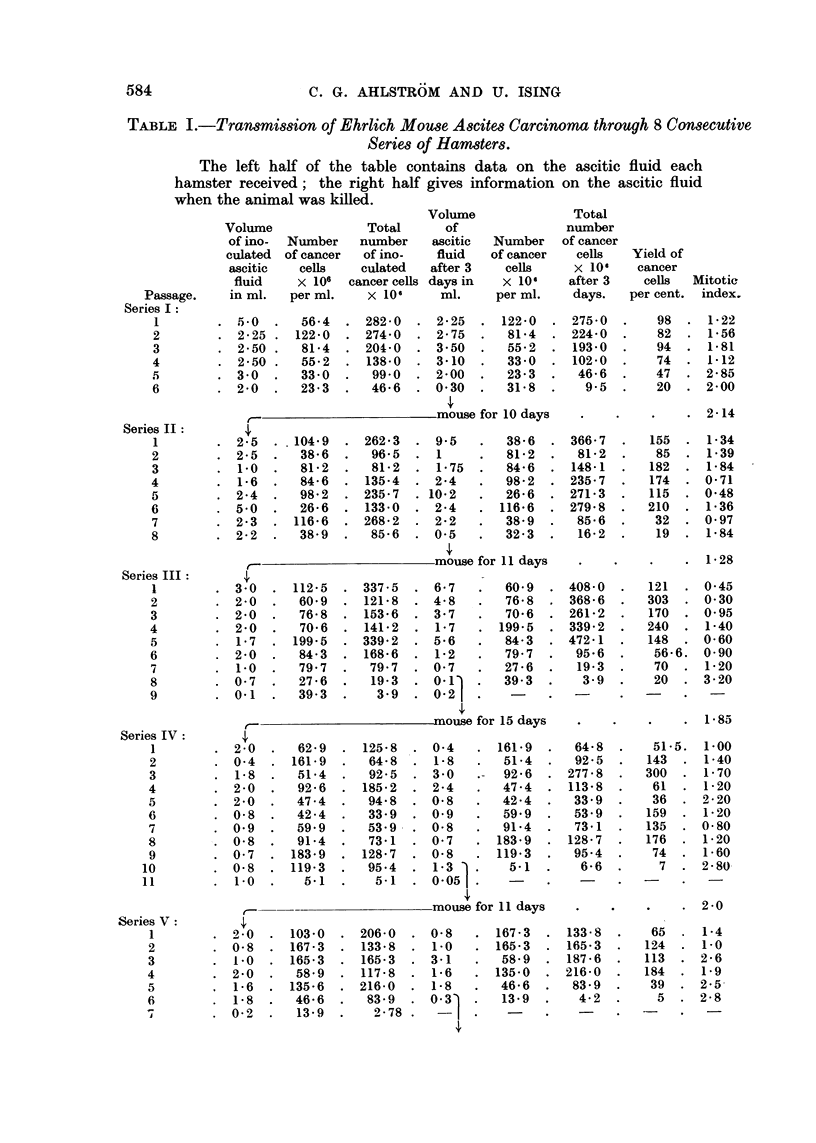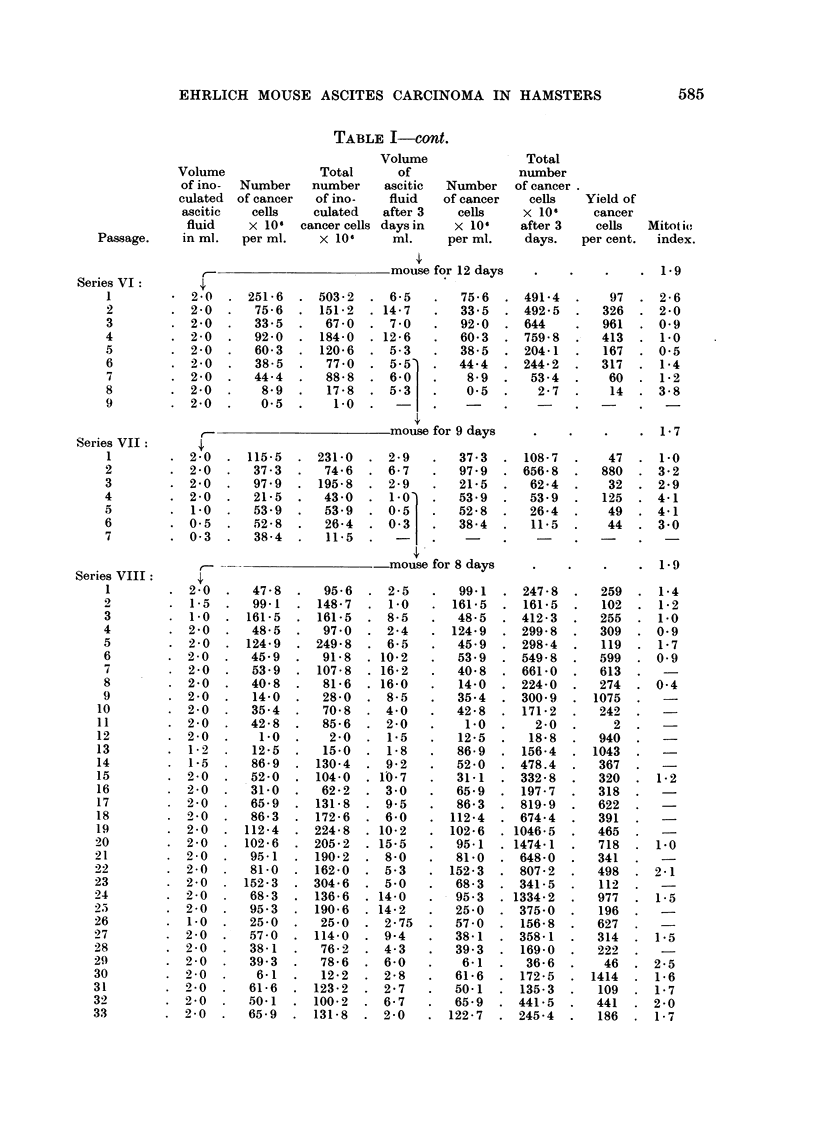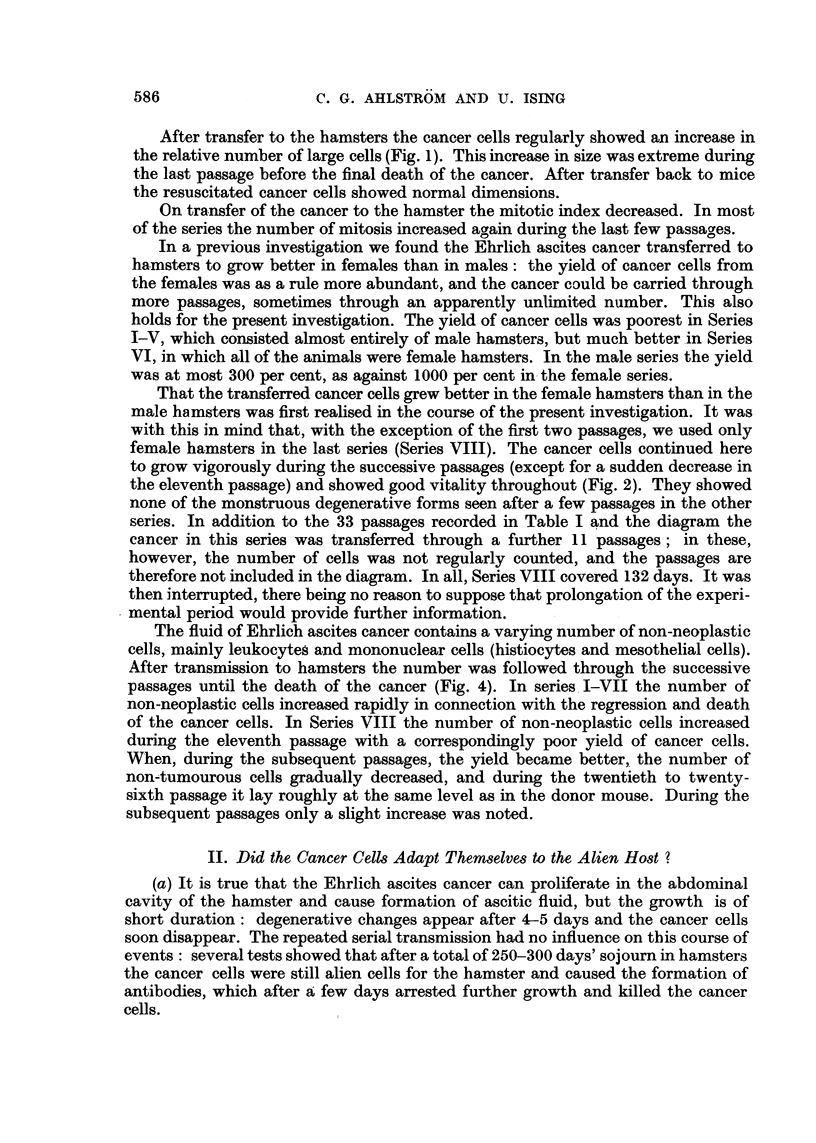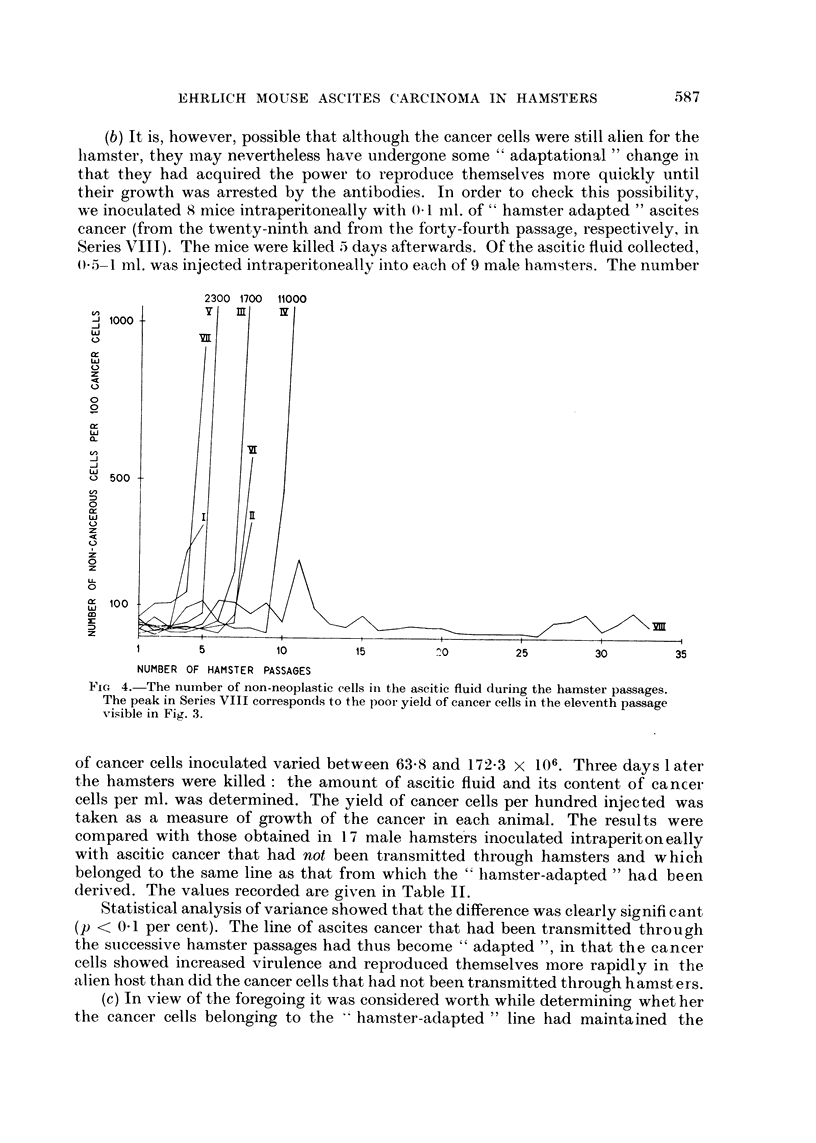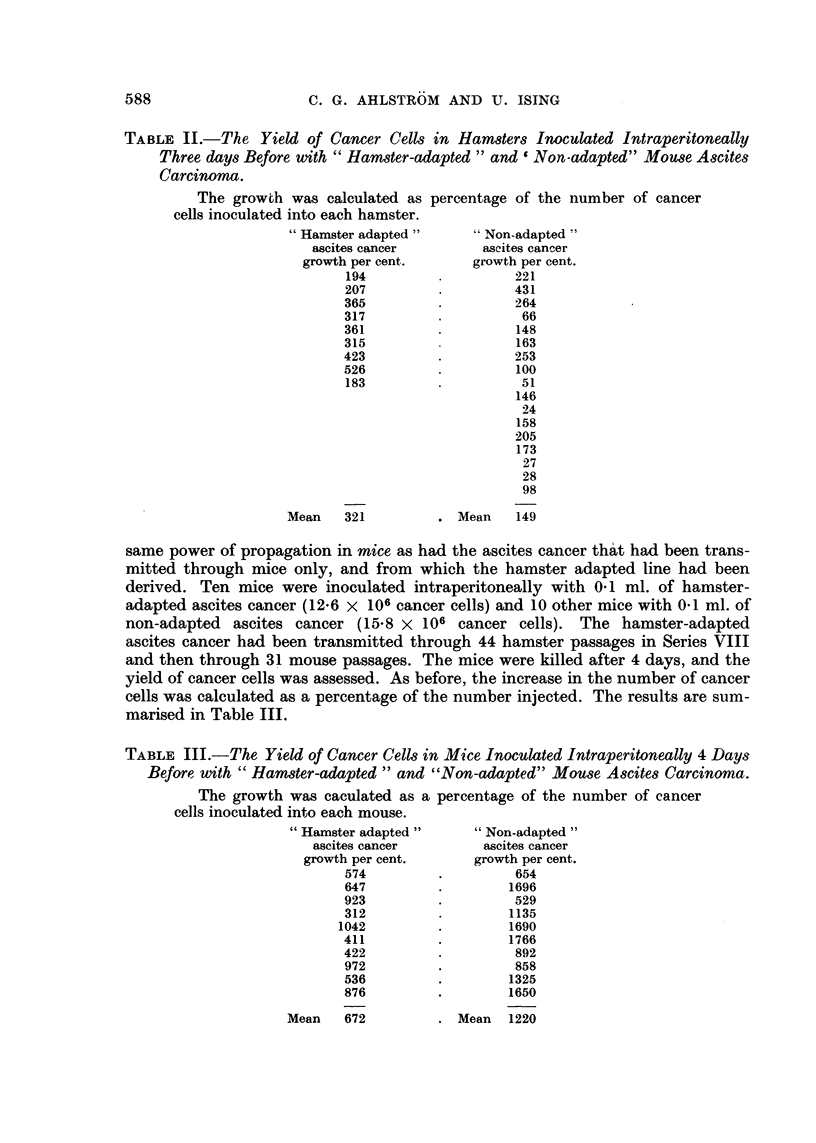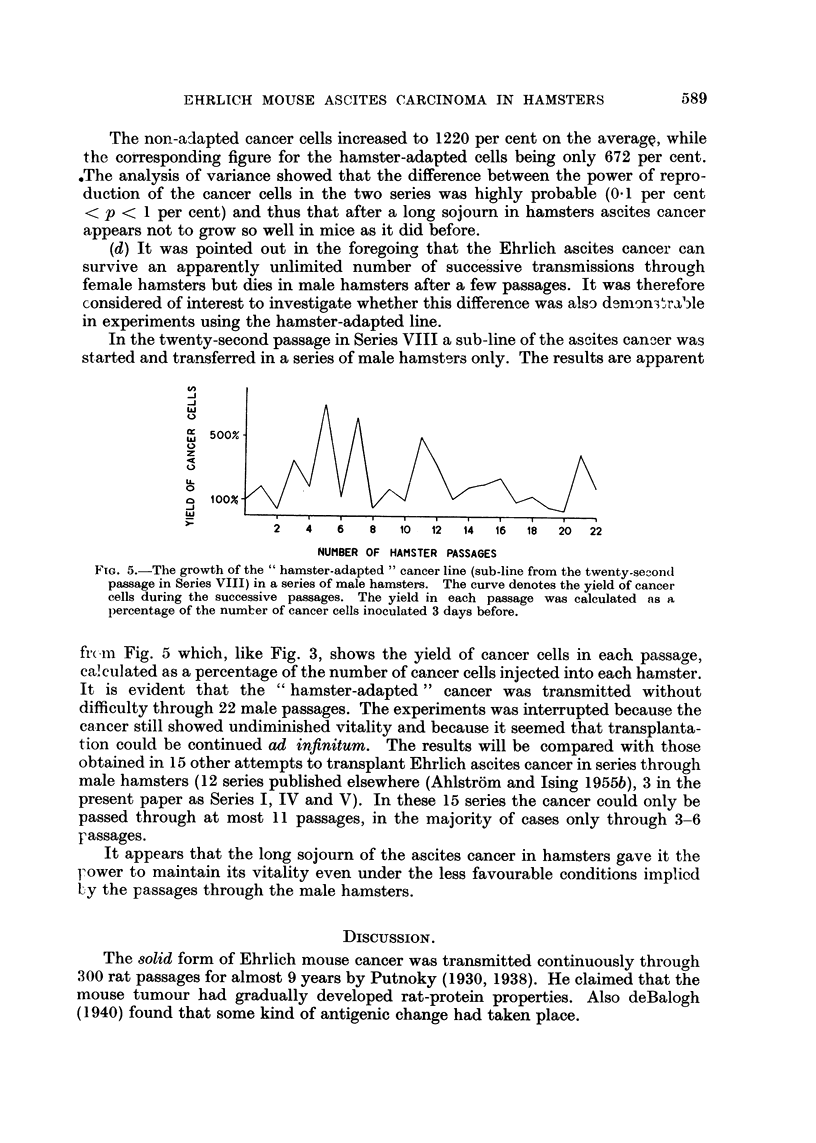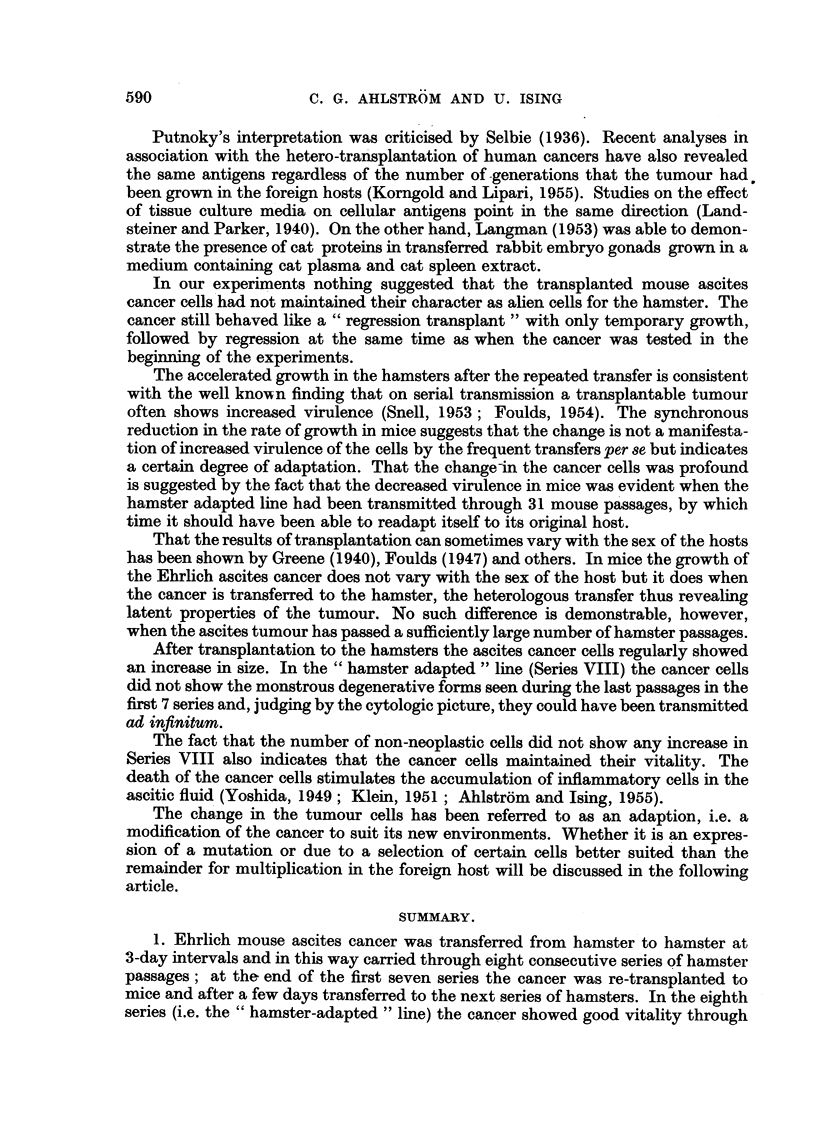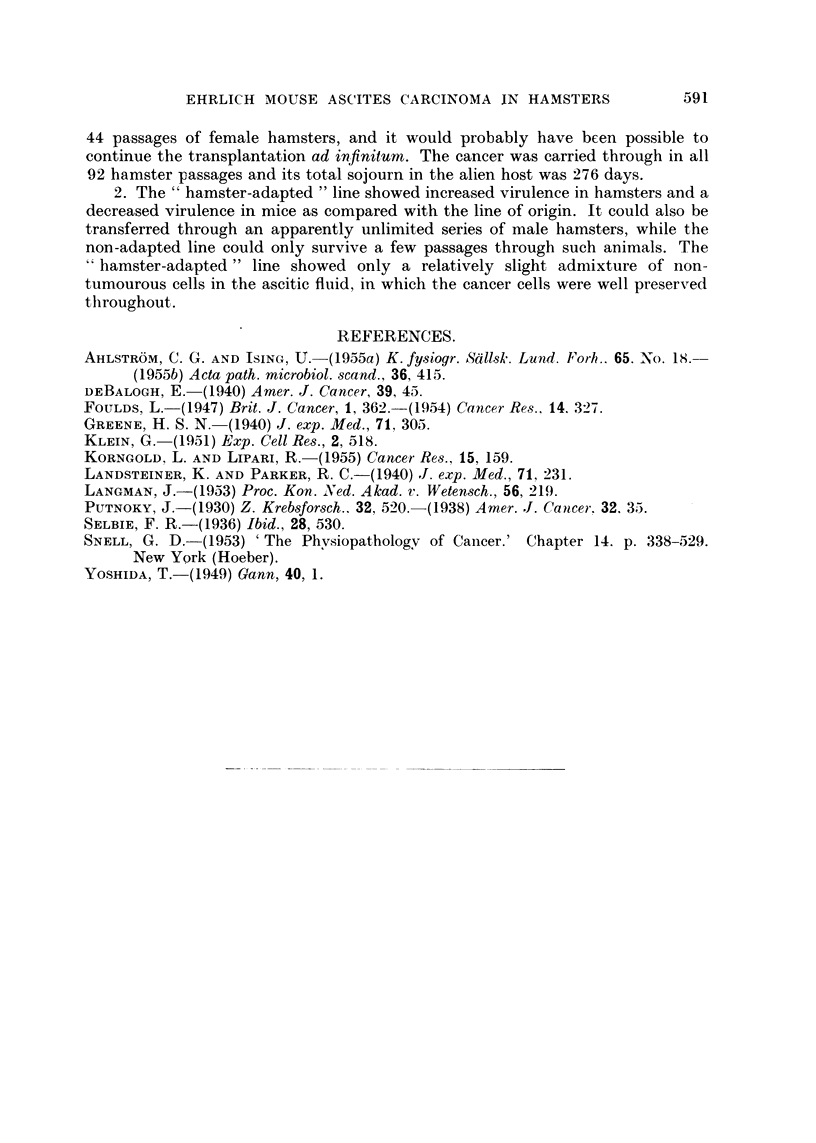# Alteration in Growth of Ehrlich Mouse Ascites Carcinoma on Repeated Transfer through Hamsters

**DOI:** 10.1038/bjc.1955.59

**Published:** 1955-12

**Authors:** C. G. Ahlström, U. Ising

## Abstract

**Images:**


### ALTERATION IN GROWTH OF EHRLICH MOUSE ASCITES CARCINOMA ON REPEATED TRANSFER THROUGH HAMSTERS.

C. G. AHLSTRÖM AND U. ISING.

*From the Institute of Pathology, University of Lund, Sweden.*

Received for publication August 12, 1955.

THE Ehrlich mouse ascites cancer can be transferred to hamsters, where it can grow with preservation of its character of ascites cancer (Ahlström and Ising, 1955). By transplantation at 3-day intervals the cancer can be transferred from hamster to hamster and survive several passages. The purpose of the present investigation was to determine whether the ascites cancer undergoes any change during successive passages through a long series of hamsters.

### MATERIAL AND METHODS.

The Ehrlich mouse ascites carcinoma was originally obtained from Dr. Georg Klein, Institute for Cell Research, Stockholm. All mice used belonged to the same inbred strain.

The experimental animals were Syrian golden hamsters weighing 100–120 g. All of the animals were brought up on a standard diet.

The first hamster in Series I was inoculated intraperitoneally with 5 ml. of pooled ascitic fluid from a batch of mice inoculated 6 days before. The hamster was killed 3 days after the inoculation. The ascitic fluid was carefully collected, measured and, after determination of its content of cancer cells, transferred to another hamster. The cancer was then transmitted in the same way at 3-day intervals to new hamsters until, after a number of passages, the cancer gave the impression that it was about to die. The ascitic fluid was then injected intraperitoneally into a few mice. The mice were killed as soon as they showed signs of ascites, and the cancer was again transferred to a hamster. It was then again carried through a series of hamsters (Series II) until the number of cancer cells in the ascitic fluid was so small that there was a risk of the cancer dying. The cancer was resuscitated by a new passage through mice, after which a new series of hamsters was started (Series III). In this way the cancer was passed through 7 series of hamsters, each series consisting of 6 to 9 passages. The last hamster series (Series VIII) was interrupted after 44 passages, since it now appeared that the ascites cancer could be transferred *ad infinitum.*

Smears were taken from each animal and stained according to Papanicolaou. The cancer cells were counted in a Buerker's haemocytometer, counting 9 squares

---

EXPLANATION OF PLATES.

FIG. 1.—Ascites cancer cells during successive passages in Series II. A : the donor mouse. B : the first hamster passage. C : the eighth hamster passage. Papanicolaou staining. × 880.

FIG. 2.—Ascites cancer cells during the successive passages in Series VIII. A : the donor mouse. B : the twenty-fifth hamster passage. C : the forty-fourth hamster passage. Papanicolaou staining. × 880.

1a

1b

1c

Ahlström and Ising.

of each chamber (each square containing 0·1 $mm^3$.). The sample of the ascitic fluid to be used for cell counting was diluted 1 : 100 or 1 : 200 with physiological saline containing 0·05 per cent eosin.

The number of non-tumourous cells in the ascitic fluid was assessed by the number found in the smears : 1000–3000 neoplastic and non-neoplastic cells were counted ; the number of leukocytes and mononuclear cells was correlated with 100 cancer cells.

The number of cancer cells in mitosis per 100 cancer cells was taken as the mitotic index. The number of cancer cells counted ranged from 1500 to 3000.

### RESULTS.

### I. *General Course of the Experiments.*

The results are given below in Table I and Fig. 3. The left half of the table contains data about the ascitic fluid each animal received. The right half of the

FIG. 3.—The yield of cancer cells in 8 consecutive series of hamster passages. The yield of cancer cells in each passage hamster was calculated as a percentage of the number of cancer cells injected 3 days before.

table gives information on the ascitic fluid at the death of the hamster 3 days later. The yield of cancer cells in each hamster, calculated as a percentage of the number injected, provides a measure of growth of the cancer cells and is given in the last column but one of the table. The diagram in Fig. 3 is based on the figures in this column. The continuous lines in the diagram denote that the host was a female hamster ; the dotted lines, that the host was a male hamster.

In the first 7 series the cancer survived only 5–10 passages. It is possible that in Series I and II the cancer cells might have been able to survive still another passage. The poorest growth of the cancer cells was seen in Series I, where the yield of cancer cells from each hamster never exceeded the amount injected. On two occasions in Series III and IV the number of cells increased to threefold the number injected. In two instances in Series VI and VII the increase was almost ten-fold.

TABLE I.—*Transmission of Ehrlich Mouse Ascites Carcinoma through* 8 *Consecutive Series of Hamsters.*

The left half of the table contains data on the ascitic fluid each hamster received; the right half gives information on the ascitic fluid when the animal was killed.

| Passage. | Volume of inoculated ascitic fluid in ml. | Number of cancer cells × $10^6$ per ml. | Total number of inoculated cancer cells × $10^6$ | Volume of ascitic fluid after 3 days in ml. | Number of cancer cells × $10^6$ per ml. | Total number of cancer cells × $10^6$ after 3 days. | Yield of cancer cells per cent. | Mitotic index. |
|---|---|---|---|---|---|---|---|---|
| Series I: | | | | | | | | |
| 1 | 5·0 | 56·4 | 282·0 | 2·25 | 122·0 | 275·0 | 98 | 1·22 |
| 2 | 2·25 | 122·0 | 274·0 | 2·75 | 81·4 | 224·0 | 82 | 1·56 |
| 3 | 2·50 | 81·4 | 204·0 | 3·50 | 55·2 | 193·0 | 94 | 1·81 |
| 4 | 2·50 | 55·2 | 138·0 | 3·10 | 33·0 | 102·0 | 74 | 1·12 |
| 5 | 3·0 | 33·0 | 99·0 | 2·00 | 23·3 | 46·6 | 47 | 2·85 |
| 6 | 2·0 | 23·3 | 46·6 | 0·30 | 31·8 | 9·5 | 20 | 2·00 |
| | | | | ↓ mouse for 10 days | | | | 2·14 |
| Series II: | | | | | | | | |
| 1 | 2·5 | 104·9 | 262·3 | 9·5 | 38·6 | 366·7 | 155 | 1·34 |
| 2 | 2·5 | 38·6 | 96·5 | 1 | 81·2 | 81·2 | 85 | 1·39 |
| 3 | 1·0 | 81·2 | 81·2 | 1·75 | 84·6 | 148·1 | 182 | 1·84 |
| 4 | 1·6 | 84·6 | 135·4 | 2·4 | 98·2 | 235·7 | 174 | 0·71 |
| 5 | 2·4 | 98·2 | 235·7 | 10·2 | 26·6 | 271·3 | 115 | 0·48 |
| 6 | 5·0 | 26·6 | 133·0 | 2·4 | 116·6 | 279·8 | 210 | 1·36 |
| 7 | 2·3 | 116·6 | 268·2 | 2·2 | 38·9 | 85·6 | 32 | 0·97 |
| 8 | 2·2 | 38·9 | 85·6 | 0·5 | 32·3 | 16·2 | 19 | 1·84 |
| | | | | ↓ mouse for 11 days | | | | 1·28 |
| Series III: | | | | | | | | |
| 1 | 3·0 | 112·5 | 337·5 | 6·7 | 60·9 | 408·0 | 121 | 0·45 |
| 2 | 2·0 | 60·9 | 121·8 | 4·8 | 76·8 | 368·6 | 303 | 0·30 |
| 3 | 2·0 | 76·8 | 153·6 | 3·7 | 70·6 | 261·2 | 170 | 0·95 |
| 4 | 2·0 | 70·6 | 141·2 | 1·7 | 199·5 | 339·2 | 240 | 1·40 |
| 5 | 1·7 | 199·5 | 339·2 | 5·6 | 84·3 | 472·1 | 148 | 0·60 |
| 6 | 2·0 | 84·3 | 168·6 | 1·2 | 79·7 | 95·6 | 56·6 | 0·90 |
| 7 | 1·0 | 79·7 | 79·7 | 0·7 | 27·6 | 19·3 | 70 | 1·20 |
| 8 | 0·7 | 27·6 | 19·3 | 0·1 | 39·3 | 3·9 | 20 | 3·20 |
| 9 | 0·1 | 39·3 | 3·9 | 0·2 | — | — | — | — |
| | | | | ↓ mouse for 15 days | | | | 1·85 |
| Series IV: | | | | | | | | |
| 1 | 2·0 | 62·9 | 125·8 | 0·4 | 161·9 | 64·8 | 51·5 | 1·00 |
| 2 | 0·4 | 161·9 | 64·8 | 1·8 | 51·4 | 92·5 | 143 | 1·40 |
| 3 | 1·8 | 51·4 | 92·5 | 3·0 | 92·6 | 277·8 | 300 | 1·70 |
| 4 | 2·0 | 92·6 | 185·2 | 2·4 | 47·4 | 113·8 | 61 | 1·20 |
| 5 | 2·0 | 47·4 | 94·8 | 0·8 | 42·4 | 33·9 | 36 | 2·20 |
| 6 | 0·8 | 42·4 | 33·9 | 0·9 | 59·9 | 53·9 | 159 | 1·20 |
| 7 | 0·9 | 59·9 | 53·9 | 0·8 | 91·4 | 73·1 | 135 | 0·80 |
| 8 | 0·8 | 91·4 | 73·1 | 0·7 | 183·9 | 128·7 | 176 | 1·20 |
| 9 | 0·7 | 183·9 | 128·7 | 0·8 | 119·3 | 95·4 | 74 | 1·60 |
| 10 | 0·8 | 119·3 | 95·4 | 1·3 | 5·1 | 6·6 | 7 | 2·80 |
| 11 | 1·0 | 5·1 | 5·1 | 0·05 | — | — | — | — |
| | | | | ↓ mouse for 11 days | | | | 2·0 |
| Series V: | | | | | | | | |
| 1 | 2·0 | 103·0 | 206·0 | 0·8 | 167·3 | 133·8 | 65 | 1·4 |
| 2 | 0·8 | 167·3 | 133·8 | 1·0 | 165·3 | 165·3 | 124 | 1·0 |
| 3 | 1·0 | 165·3 | 165·3 | 3·1 | 58·9 | 187·6 | 113 | 2·6 |
| 4 | 2·0 | 58·9 | 117·8 | 1·6 | 135·0 | 216·0 | 184 | 1·9 |
| 5 | 1·6 | 135·6 | 216·0 | 1·8 | 46·6 | 83·9 | 39 | 2·5 |
| 6 | 1·8 | 46·6 | 83·9 | 0·3 | 13·9 | 4·2 | 5 | 2·8 |
| 7 | 0·2 | 13·9 | 2·78 | — | — | — | — | — |
| | | | | ↓ | | | | |

TABLE I—*cont.*

| Passage. | Volume of ino-culated ascitic fluid in ml. | Number of cancer cells × $10^6$ per ml. | Total number of ino-culated cancer cells × $10^6$ | Volume of ascitic fluid after 3 days in ml. | Number of cancer cells × $10^6$ per ml. | Total number of cancer cells × $10^6$ after 3 days. | Yield of cancer cells per cent. | Mitotic index. |
|---|---|---|---|---|---|---|---|---|
| | | | | mouse for 12 days | | | | 1·9 |
| Series VI : | | | | | | | | |
| 1 | 2·0 | 251·6 | 503·2 | 6·5 | 75·6 | 491·4 | 97 | 2·6 |
| 2 | 2·0 | 75·6 | 151·2 | 14·7 | 33·5 | 492·5 | 326 | 2·0 |
| 3 | 2·0 | 33·5 | 67·0 | 7·0 | 92·0 | 644 | 961 | 0·9 |
| 4 | 2·0 | 92·0 | 184·0 | 12·6 | 60·3 | 759·8 | 413 | 1·0 |
| 5 | 2·0 | 60·3 | 120·6 | 5·3 | 38·5 | 204·1 | 167 | 0·5 |
| 6 | 2·0 | 38·5 | 77·0 | 5·5 | 44·4 | 244·2 | 317 | 1·4 |
| 7 | 2·0 | 44·4 | 88·8 | 6·0 | 8·9 | 53·4 | 60 | 1·2 |
| 8 | 2·0 | 8·9 | 17·8 | 5·3 | 0·5 | 2·7 | 14 | 3·8 |
| 9 | 2·0 | 0·5 | 1·0 | — | — | — | — | — |
| | | | | mouse for 9 days | | | | 1·7 |
| Series VII : | | | | | | | | |
| 1 | 2·0 | 115·5 | 231·0 | 2·9 | 37·3 | 108·7 | 47 | 1·0 |
| 2 | 2·0 | 37·3 | 74·6 | 6·7 | 97·9 | 656·8 | 880 | 3·2 |
| 3 | 2·0 | 97·9 | 195·8 | 2·9 | 21·5 | 62·4 | 32 | 2·9 |
| 4 | 2·0 | 21·5 | 43·0 | 1·0 | 53·9 | 53·9 | 125 | 4·1 |
| 5 | 1·0 | 53·9 | 53·9 | 0·5 | 52·8 | 26·4 | 49 | 4·1 |
| 6 | 0·5 | 52·8 | 26·4 | 0·3 | 38·4 | 11·5 | 44 | 3·0 |
| 7 | 0·3 | 38·4 | 11·5 | — | — | — | — | — |
| | | | | mouse for 8 days | | | | 1·9 |
| Series VIII : | | | | | | | | |
| 1 | 2·0 | 47·8 | 95·6 | 2·5 | 99·1 | 247·8 | 259 | 1·4 |
| 2 | 1·5 | 99·1 | 148·7 | 1·0 | 161·5 | 161·5 | 102 | 1·2 |
| 3 | 1·0 | 161·5 | 161·5 | 8·5 | 48·5 | 412·3 | 255 | 1·0 |
| 4 | 2·0 | 48·5 | 97·0 | 2·4 | 124·9 | 299·8 | 309 | 0·9 |
| 5 | 2·0 | 124·9 | 249·8 | 6·5 | 45·9 | 298·4 | 119 | 1·7 |
| 6 | 2·0 | 45·9 | 91·8 | 10·2 | 53·9 | 549·8 | 599 | 0·9 |
| 7 | 2·0 | 53·9 | 107·8 | 16·2 | 40·8 | 661·0 | 613 | — |
| 8 | 2·0 | 40·8 | 81·6 | 16·0 | 14·0 | 224·0 | 274 | 0·4 |
| 9 | 2·0 | 14·0 | 28·0 | 8·5 | 35·4 | 300·9 | 1075 | — |
| 10 | 2·0 | 35·4 | 70·8 | 4·0 | 42·8 | 171·2 | 242 | — |
| 11 | 2·0 | 42·8 | 85·6 | 2·0 | 1·0 | 2·0 | 2 | — |
| 12 | 2·0 | 1·0 | 2·0 | 1·5 | 12·5 | 18·8 | 940 | — |
| 13 | 1·2 | 12·5 | 15·0 | 1·8 | 86·9 | 156·4 | 1043 | — |
| 14 | 1·5 | 86·9 | 130·4 | 9·2 | 52·0 | 478.4 | 367 | — |
| 15 | 2·0 | 52·0 | 104·0 | 10·7 | 31·1 | 332·8 | 320 | 1·2 |
| 16 | 2·0 | 31·0 | 62·2 | 3·0 | 65·9 | 197·7 | 318 | — |
| 17 | 2·0 | 65·9 | 131·8 | 9·5 | 86·3 | 819·9 | 622 | — |
| 18 | 2·0 | 86·3 | 172·6 | 6·0 | 112·4 | 674·4 | 391 | — |
| 19 | 2·0 | 112·4 | 224·8 | 10·2 | 102·6 | 1046·5 | 465 | — |
| 20 | 2·0 | 102·6 | 205·2 | 15·5 | 95·1 | 1474·1 | 718 | 1·0 |
| 21 | 2·0 | 95·1 | 190·2 | 8·0 | 81·0 | 648·0 | 341 | — |
| 22 | 2·0 | 81·0 | 162·0 | 5·3 | 152·3 | 807·2 | 498 | 2·1 |
| 23 | 2·0 | 152·3 | 304·6 | 5·0 | 68·3 | 341·5 | 112 | — |
| 24 | 2·0 | 68·3 | 136·6 | 14·0 | 95·3 | 1334·2 | 977 | 1·5 |
| 25 | 2·0 | 95·3 | 190·6 | 14·2 | 25·0 | 375·0 | 196 | — |
| 26 | 1·0 | 25·0 | 25·0 | 2·75 | 57·0 | 156·8 | 627 | — |
| 27 | 2·0 | 57·0 | 114·0 | 9·4 | 38·1 | 358·1 | 314 | 1·5 |
| 28 | 2·0 | 38·1 | 76·2 | 4·3 | 39·3 | 169·0 | 222 | — |
| 29 | 2·0 | 39·3 | 78·6 | 6·0 | 6·1 | 36·6 | 46 | 2·5 |
| 30 | 2·0 | 6·1 | 12·2 | 2·8 | 61·6 | 172·5 | 1414 | 1·6 |
| 31 | 2·0 | 61·6 | 123·2 | 2·7 | 50·1 | 135·3 | 109 | 1·7 |
| 32 | 2·0 | 50·1 | 100·2 | 6·7 | 65·9 | 441·5 | 441 | 2·0 |
| 33 | 2·0 | 65·9 | 131·8 | 2·0 | 122·7 | 245·4 | 186 | 1·7 |

After transfer to the hamsters the cancer cells regularly showed an increase in the relative number of large cells (Fig. 1). This increase in size was extreme during the last passage before the final death of the cancer. After transfer back to mice the resuscitated cancer cells showed normal dimensions.

On transfer of the cancer to the hamster the mitotic index decreased. In most of the series the number of mitosis increased again during the last few passages.

In a previous investigation we found the Ehrlich ascites cancer transferred to hamsters to grow better in females than in males : the yield of cancer cells from the females was as a rule more abundant, and the cancer could be carried through more passages, sometimes through an apparently unlimited number. This also holds for the present investigation. The yield of cancer cells was poorest in Series I–V, which consisted almost entirely of male hamsters, but much better in Series VI, in which all of the animals were female hamsters. In the male series the yield was at most 300 per cent, as against 1000 per cent in the female series.

That the transferred cancer cells grew better in the female hamsters than in the male hamsters was first realised in the course of the present investigation. It was with this in mind that, with the exception of the first two passages, we used only female hamsters in the last series (Series VIII). The cancer cells continued here to grow vigorously during the successive passages (except for a sudden decrease in the eleventh passage) and showed good vitality throughout (Fig. 2). They showed none of the monstruous degenerative forms seen after a few passages in the other series. In addition to the 33 passages recorded in Table I and the diagram the cancer in this series was transferred through a further 11 passages ; in these, however, the number of cells was not regularly counted, and the passages are therefore not included in the diagram. In all, Series VIII covered 132 days. It was then interrupted, there being no reason to suppose that prolongation of the experimental period would provide further information.

The fluid of Ehrlich ascites cancer contains a varying number of non-neoplastic cells, mainly leukocytes and mononuclear cells (histiocytes and mesothelial cells). After transmission to hamsters the number was followed through the successive passages until the death of the cancer (Fig. 4). In series I–VII the number of non-neoplastic cells increased rapidly in connection with the regression and death of the cancer cells. In Series VIII the number of non-neoplastic cells increased during the eleventh passage with a correspondingly poor yield of cancer cells. When, during the subsequent passages, the yield became better, the number of non-tumourous cells gradually decreased, and during the twentieth to twenty-sixth passage it lay roughly at the same level as in the donor mouse. During the subsequent passages only a slight increase was noted.

### II. *Did the Cancer Cells Adapt Themselves to the Alien Host ?*

(*a*) It is true that the Ehrlich ascites cancer can proliferate in the abdominal cavity of the hamster and cause formation of ascitic fluid, but the growth is of short duration : degenerative changes appear after 4–5 days and the cancer cells soon disappear. The repeated serial transmission had no influence on this course of events : several tests showed that after a total of 250–300 days' sojourn in hamsters the cancer cells were still alien cells for the hamster and caused the formation of antibodies, which after a few days arrested further growth and killed the cancer cells.

(*b*) It is, however, possible that although the cancer cells were still alien for the hamster, they may nevertheless have undergone some " adaptational " change in that they had acquired the power to reproduce themselves more quickly until their growth was arrested by the antibodies. In order to check this possibility, we inoculated 8 mice intraperitoneally with 0·1 ml. of " hamster adapted " ascites cancer (from the twenty-ninth and from the forty-fourth passage, respectively, in Series VIII). The mice were killed 5 days afterwards. Of the ascitic fluid collected, 0·5–1 ml. was injected intraperitoneally into each of 9 male hamsters. The number

FIG 4.—The number of non-neoplastic cells in the ascitic fluid during the hamster passages. The peak in Series VIII corresponds to the poor yield of cancer cells in the eleventh passage visible in Fig. 3.

of cancer cells inoculated varied between 63·8 and 172·3 $\times$ $10^6$. Three days later the hamsters were killed : the amount of ascitic fluid and its content of cancer cells per ml. was determined. The yield of cancer cells per hundred injected was taken as a measure of growth of the cancer in each animal. The results were compared with those obtained in 17 male hamsters inoculated intraperitoneally with ascitic cancer that had *not* been transmitted through hamsters and which belonged to the same line as that from which the " hamster-adapted " had been derived. The values recorded are given in Table II.

Statistical analysis of variance showed that the difference was clearly significant ($p < 0·1$ per cent). The line of ascites cancer that had been transmitted through the successive hamster passages had thus become " adapted ", in that the cancer cells showed increased virulence and reproduced themselves more rapidly in the alien host than did the cancer cells that had not been transmitted through hamsters.

(*c*) In view of the foregoing it was considered worth while determining whether the cancer cells belonging to the " hamster-adapted " line had maintained the

TABLE II.—*The Yield of Cancer Cells in Hamsters Inoculated Intraperitoneally Three days Before with " Hamster-adapted " and ' Non-adapted" Mouse Ascites Carcinoma.*

The growth was calculated as percentage of the number of cancer cells inoculated into each hamster.

| " Hamster adapted " ascites cancer growth per cent. | " Non-adapted " ascites cancer growth per cent. |
|---|---|
| 194 | 221 |
| 207 | 431 |
| 365 | 264 |
| 317 | 66 |
| 361 | 148 |
| 315 | 163 |
| 423 | 253 |
| 526 | 100 |
| 183 | 51 |
| | 146 |
| | 24 |
| | 158 |
| | 205 |
| | 173 |
| | 27 |
| | 28 |
| | 98 |
| Mean 321 | Mean 149 |

same power of propagation in *mice* as had the ascites cancer that had been transmitted through mice only, and from which the hamster adapted line had been derived. Ten mice were inoculated intraperitoneally with 0·1 ml. of hamster-adapted ascites cancer ($12 \cdot 6 \times 10^6$ cancer cells) and 10 other mice with 0·1 ml. of non-adapted ascites cancer ($15 \cdot 8 \times 10^6$ cancer cells). The hamster-adapted ascites cancer had been transmitted through 44 hamster passages in Series VIII and then through 31 mouse passages. The mice were killed after 4 days, and the yield of cancer cells was assessed. As before, the increase in the number of cancer cells was calculated as a percentage of the number injected. The results are summarised in Table III.

TABLE III.—*The Yield of Cancer Cells in Mice Inoculated Intraperitoneally* 4 *Days Before with " Hamster-adapted " and "Non-adapted" Mouse Ascites Carcinoma.*

The growth was caculated as a percentage of the number of cancer cells inoculated into each mouse.

| " Hamster adapted " ascites cancer growth per cent. | " Non-adapted " ascites cancer growth per cent. |
|---|---|
| 574 | 654 |
| 647 | 1696 |
| 923 | 529 |
| 312 | 1135 |
| 1042 | 1690 |
| 411 | 1766 |
| 422 | 892 |
| 972 | 858 |
| 536 | 1325 |
| 876 | 1650 |
| Mean 672 | Mean 1220 |

The non-adapted cancer cells increased to 1220 per cent on the average, while the corresponding figure for the hamster-adapted cells being only 672 per cent. The analysis of variance showed that the difference between the power of reproduction of the cancer cells in the two series was highly probable (0·1 per cent $< p <$ 1 per cent) and thus that after a long sojourn in hamsters ascites cancer appears not to grow so well in mice as it did before.

(*d*) It was pointed out in the foregoing that the Ehrlich ascites cancer can survive an apparently unlimited number of successive transmissions through female hamsters but dies in male hamsters after a few passages. It was therefore considered of interest to investigate whether this difference was also demonstrable in experiments using the hamster-adapted line.

In the twenty-second passage in Series VIII a sub-line of the ascites cancer was started and transferred in a series of male hamsters only. The results are apparent

FIG. 5.—The growth of the " hamster-adapted " cancer line (sub-line from the twenty-second passage in Series VIII) in a series of male hamsters. The curve denotes the yield of cancer cells during the successive passages. The yield in each passage was calculated as a percentage of the number of cancer cells inoculated 3 days before.

from Fig. 5 which, like Fig. 3, shows the yield of cancer cells in each passage, calculated as a percentage of the number of cancer cells injected into each hamster. It is evident that the " hamster-adapted " cancer was transmitted without difficulty through 22 male passages. The experiments was interrupted because the cancer still showed undiminished vitality and because it seemed that transplantation could be continued *ad infinitum*. The results will be compared with those obtained in 15 other attempts to transplant Ehrlich ascites cancer in series through male hamsters (12 series published elsewhere (Ahlström and Ising 1955*b*), 3 in the present paper as Series I, IV and V). In these 15 series the cancer could only be passed through at most 11 passages, in the majority of cases only through 3–6 passages.

It appears that the long sojourn of the ascites cancer in hamsters gave it the power to maintain its vitality even under the less favourable conditions implied by the passages through the male hamsters.

### DISCUSSION.

The *solid* form of Ehrlich mouse cancer was transmitted continuously through 300 rat passages for almost 9 years by Putnoky (1930, 1938). He claimed that the mouse tumour had gradually developed rat-protein properties. Also deBalogh (1940) found that some kind of antigenic change had taken place.

Putnoky's interpretation was criticised by Selbie (1936). Recent analyses in association with the hetero-transplantation of human cancers have also revealed the same antigens regardless of the number of generations that the tumour had been grown in the foreign hosts (Korngold and Lipari, 1955). Studies on the effect of tissue culture media on cellular antigens point in the same direction (Landsteiner and Parker, 1940). On the other hand, Langman (1953) was able to demonstrate the presence of cat proteins in transferred rabbit embryo gonads grown in a medium containing cat plasma and cat spleen extract.

In our experiments nothing suggested that the transplanted mouse ascites cancer cells had not maintained their character as alien cells for the hamster. The cancer still behaved like a " regression transplant " with only temporary growth, followed by regression at the same time as when the cancer was tested in the beginning of the experiments.

The accelerated growth in the hamsters after the repeated transfer is consistent with the well known finding that on serial transmission a transplantable tumour often shows increased virulence (Snell, 1953 ; Foulds, 1954). The synchronous reduction in the rate of growth in mice suggests that the change is not a manifestation of increased virulence of the cells by the frequent transfers *per se* but indicates a certain degree of adaptation. That the change in the cancer cells was profound is suggested by the fact that the decreased virulence in mice was evident when the hamster adapted line had been transmitted through 31 mouse passages, by which time it should have been able to readapt itself to its original host.

That the results of transplantation can sometimes vary with the sex of the hosts has been shown by Greene (1940), Foulds (1947) and others. In mice the growth of the Ehrlich ascites cancer does not vary with the sex of the host but it does when the cancer is transferred to the hamster, the heterologous transfer thus revealing latent properties of the tumour. No such difference is demonstrable, however, when the ascites tumour has passed a sufficiently large number of hamster passages.

After transplantation to the hamsters the ascites cancer cells regularly showed an increase in size. In the " hamster adapted " line (Series VIII) the cancer cells did not show the monstrous degenerative forms seen during the last passages in the first 7 series and, judging by the cytologic picture, they could have been transmitted *ad infinitum*.

The fact that the number of non-neoplastic cells did not show any increase in Series VIII also indicates that the cancer cells maintained their vitality. The death of the cancer cells stimulates the accumulation of inflammatory cells in the ascitic fluid (Yoshida, 1949 ; Klein, 1951 ; Ahlström and Ising, 1955).

The change in the tumour cells has been referred to as an adaption, i.e. a modification of the cancer to suit its new environments. Whether it is an expression of a mutation or due to a selection of certain cells better suited than the remainder for multiplication in the foreign host will be discussed in the following article.

### SUMMARY.

1. Ehrlich mouse ascites cancer was transferred from hamster to hamster at 3-day intervals and in this way carried through eight consecutive series of hamster passages ; at the end of the first seven series the cancer was re-transplanted to mice and after a few days transferred to the next series of hamsters. In the eighth series (i.e. the " hamster-adapted " line) the cancer showed good vitality through